# Understanding cognitive control in aging: A brain network perspective

**DOI:** 10.3389/fnagi.2022.1038756

**Published:** 2022-10-31

**Authors:** Haishuo Xia, Qinghua He, Antao Chen

**Affiliations:** ^1^Faculty of Psychology, Southwest University, Chongqing, China; ^2^School of Psychology, Shanghai University of Sport, Shanghai, China

**Keywords:** aging, cognitive control, functional connectivity, functional network, neural network mechanisms, structural network, executive function

## Abstract

Cognitive control decline is a major manifestation of brain aging that severely impairs the goal-directed abilities of older adults. Magnetic resonance imaging evidence suggests that cognitive control during aging is associated with altered activation in a range of brain regions, including the frontal, parietal, and occipital lobes. However, focusing on specific regions, while ignoring the structural and functional connectivity between regions, may impede an integrated understanding of cognitive control decline in older adults. Here, we discuss the role of aging-related changes in functional segregation, integration, and antagonism among large-scale networks. We highlight that disrupted spontaneous network organization, impaired information co-processing, and enhanced endogenous interference promote cognitive control declines during aging. Additionally, in older adults, severe damage to structural network can weaken functional connectivity and subsequently trigger cognitive control decline, whereas a relatively intact structural network ensures the compensation of functional connectivity to mitigate cognitive control impairment. Thus, we propose that age-related changes in functional networks may be influenced by structural networks in cognitive control in aging (CCA). This review provided an integrative framework to understand the cognitive control decline in aging by viewing the brain as a multimodal networked system.

## Introduction

Over the past 20 years, the proportion of older adults in the population has expanded rapidly (Beard et al., 2016; He et al., 2016). Advances in healthcare have delayed the debilitating effects of aging on physical wellbeing (Czaja et al., 2013). Nevertheless, striking declines in measures of cognitive control continue to be associated with aging (Grady, 2012). Cognitive control (also termed executive function) refers to several cognitive processes involved in top-down control, including inhibitory control, working memory, and cognitive flexibility (Diamond, 2013; Zink et al., 2021). Miyake et al. (2000) also explicitly addressed the diversity of cognitive control and proposed three distinct processes: updating (or monitoring), inhibition, and switching (or shifting). Despite the diversity, these control-based cognitions rely on “dampening” irrelevant information and prioritizing relevant information to ensure goal-oriented tasks (Gratton et al., 2018). In addition, cognitive control supports a series of higher-order cognitive processes (e.g., planning and reasoning) and is needed to meet the demands of daily life (Diamond, 2013). Accordingly, cognitive control in aging (CCA) may involve a decline in overall cognition. Given that cognitive declines impair the quality of life and life satisfaction in older adults (Jones et al., 2003; Khodabakhsh, 2021), it is valuable to understand the neurological mechanisms of CCA, by which we could facilitate the development of interventions that can address brain aging (Williams and Kemper, 2010).

To date, a major focus in neurocognitive aging research was linking cognitive control declines to specific regions (Coxon et al., 2016; Fernandez-Ruiz et al., 2018). From this perspective, CCA emerges from the functional degeneration of discrete regions. For example, aging is associated with decreased activation of occipital regions (Spreng et al., 2010; Li et al., 2015), suggesting that CCA may begin with a decline in visual perception. Moreover, older adults show increased or decreased activation in the frontal and parietal regions during tasks requiring cognitive control (Cappell et al., 2010; Spreng et al., 2017). The compensation-related utilization of neural circuits hypothesis (CRUNCH) proposes that older adults typically utilize more neural resources to meet task demands and exhibit hyperactivation under low-demand conditions (Reuter-Lorenz and Lustig, 2005; Reuter-Lorenz and Cappell, 2008; Festini et al., 2018). However, such a strategy fails under high-demand conditions, resulting in lower regional activation (Cappell et al., 2010; Schneider-Garces et al., 2010). In addition, CRUNCH also suggests increased bilateral recruitment of the prefrontal cortex. Specifically, older adults showed bilateral recruitment of the prefrontal cortex in both low- and high-demand conditions, while young adults recruited the bilateral prefrontal cortex only in the high-demand condition (Spaniol and Grady, 2012). Previous studies have partially revealed the neural basis of CCA by localizing regions (Cappell et al., 2010; Spaniol and Grady, 2012; Spreng et al., 2017), but this approach cannot reveal the mechanism at a systems level.

Accumulating evidence has suggested that CCA can arise from changes in connectivity among brain networks (Hausman et al., 2020; Rieck et al., 2021; Setton et al., 2021). Network neuroscience views the human brain as a complex networked system. This system is composed of several large-scale networks that enable specific mental functions (Sporns, 2013; Sporns and Betzel, 2016). Moreover, functional interactions among large-scale networks are crucial for complex cognitive control (Cocchi et al., 2013). Hence, disrupted network interactions can lead to aging-related declines in cognitive control (Grady et al., 2016; Setton et al., 2021). For example, functional networks typically show functional segregation in the resting state, which implies that the intra-network connectivity is dense while inter-network connectivity is sparse (Sporns, 2013; Wig, 2017). When the brain enters the task state, functional networks present functional integration, which is characterized by dynamic rewiring, with enhanced functional connectivity or network efficiency (Cocchi et al., 2013; Sporns, 2013). Abnormalities in functional segregation and integration among networks are associated with cognitive control declines in older adults (Grady et al., 2016; Hausman et al., 2020; Setton et al., 2021). Although aging-related changes in functional network have been observed in older adults, the structural basis of these functional changes remains to be fully investigated, and no review has discussed how changes in multimodal networks are involved in CCA. Herein, we review functional and structural neuroimaging studies to provide a systematic outline of the network mechanisms underlying CCA.

## Connectivity changes in functional networks associated with cognitive control in aging

Multiple large-scale networks facilitate specialized mental functioning for cognitive control (Cocchi et al., 2013; Sporns and Betzel, 2016). These networks can be functionally classified into three categories: the task-positive network (TPN), the default mode network (DMN), and networks involved in primary mental processes. First, the TPN is thought to be critical for complex cognitive processes (e.g., top-down control and external attention), which mainly involves the frontoparietal, dorsal attention, and ventral attention networks (Cocchi et al., 2013; Di and Biswal, 2014; Hsu et al., 2020; Yao et al., 2020). Specifically, the frontoparietal network, with the dorsolateral prefrontal cortex (dlPFC) and posterior parietal lobe (PPC) as hub regions, typically shows increased activation during cognitive control tasks and putatively in service of conflict monitoring and resolution (Qiao et al., 2017; Chen et al., 2018; Yin et al., 2018). In addition, ventral and dorsal attention networks are engaged in capturing salient stimuli and attentional control (Vossel et al., 2014; Tamber-Rosenau et al., 2018; Suo et al., 2021). Second, the DMN, also called the task-negative network, is thought to support self-reflective and internally directed cognitions (Anticevic et al., 2012; Raichle, 2015). The DMN normally shows reduced activity during exogenous cognitive demands, putatively in service of allowing a focus on external task demands (Harrison et al., 2008; Buckner and DiNicola, 2019). Third, the networks involved in primary mental processes also support cognitive control. The visual network, mainly comprising the occipital cortex, is associated with the early visual perception of task-related stimuli (Yeo et al., 2011; Cocchi et al., 2013), while the sensorimotor network is usually responsible for controlling hand movements during the late stage of reactive control (Levy and Wagner, 2011; Cocchi et al., 2013). There is evidence that age-related changes in the interaction patterns of these networks play a role in CCA.

### Decreased functional segregation in the resting state

**FIGURE 1:**
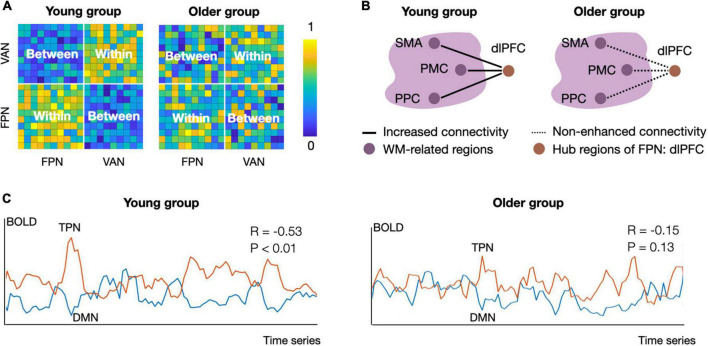
Schematic diagram of group differences in functional segregation, integration, and antagonism between young and older adults. **(A)** Functional segregation of the frontoparietal network (FPN) and ventral attention network (VAN) in the resting state. The intra-network connectivity in the young group is tightly connected, but the inter-network connectivity is weak. The connectivity pattern changes in older adults, showing decreased intra-connectivity and increased inter-connectivity of the FPN and VAN, suggesting decreased functional segregation of the task positive network (Chan et al., 2014; Zonneveld et al., 2019). **(B)** Functional integration in task processing. During the N-back task, the young group showed increased connectivity between the FPN hub (i.e., the left dlPFC) and working-memory-related regions from 1-back to 3-back conditions. Nevertheless, older adults did not show load-dependent connectivity enhancement between these regions, suggesting a decline in functional integration during aging (Nagel et al., 2011). The solid lines reflect increased connectivity strength, and the dotted lines indicate no load-dependent connectivity enhancement. Working-memory-related regions were located in the big bubble with purple color. **(C)** Functional antagonism between the TPN and DMN. The mean activity patterns of the TPN and DMN are predominantly negatively correlated in the younger group, while this correlation is absent in the older group. Panel A and C used simulated data to illustrate published findings (Nagel et al., 2011; Chan et al., 2014; Geerligs et al., 2015; Ferreira et al., 2016; Zonneveld et al., 2019). The connectivity matrix in panel **(A)** and connectivity strength in panel **(C)** were calculated by Pearson correlation. Within, intra-network connectivity; Between, inter-network connectivity; DMN, default mode network; TPN, task positive network; FPN, frontoparietal network; VAN, ventral attention network; SMA, supplementary motor area; PMC, premotor cortex; PPC, posterior parietal cortex; dlPFC, dorsolateral prefrontal cortex; WM, working memory.

Decreased functional segregation occurs with aging (Chan et al., 2014; Damoiseaux, 2017; Oschmann and Gawryluk, 2020). Functional segregation typically refers to neural processing in regions with similar functions, which usually manifests as sparse inter-network connectivity and dense intra-network connectivity (Sporns, 2013; Sporns and Betzel, 2016). In older adults, impaired functional segregation (as illustrated in Figure 1A) in the resting state occurs in the TPN and is generally characterized by decreased intra-network connectivity and increased inter-network connectivity (Damoiseaux, 2017; Oschmann and Gawryluk, 2020). For example, compared with young adults, older adults have reduced functional connectivity strength within the ventral and dorsal attentional networks (Archer et al., 2016; Zonneveld et al., 2019). Also, over a 4-year follow-up period, older adults showed a gradual decline in frontoparietal network connectivity (Oschmann and Gawryluk, 2020). In terms of inter-network connectivity, compared to young adults, older adults have increased functional connectivity between the frontoparietal and dorsal attention networks, between the frontoparietal and ventral attention networks, and between dorsal and ventral attention networks (Ferreira and Busatto, 2013; Ferreira et al., 2016; Zonneveld et al., 2019). Taken together, there is a clear association between decreased functional segregation in the TPN and aging.

Preliminary evidence has suggested that decreased functional segregation of the TPN is associated with a decline in cognitive control with age. For example, in older adults, lower intra-connectivity within frontoparietal and attention network indicates worse cognitive control, and enhanced inter-connectivity between the frontoparietal and dorsal attention networks is associated with cognitive control decline (Sala-Llonch et al., 2012; Hausman et al., 2020; Setton et al., 2021). Functional segregation reflects the extent to which densely connected regions perform specialized cognitive functions (Wig, 2017; Koen and Rugg, 2019) and often indicate a well-organized spontaneous network. Decreased functional segregation might reflect an impairment in spontaneous network organization, which in turn manifests as cognitive control decline in older adults.

### Declined functional integration during tasks requiring cognitive control

Functional integration (also called functional coupling) reflects the integrated processing of information during tasks and usually manifests as enhanced functional connectivity (Fornito et al., 2012; Cocchi et al., 2013; Sporns, 2013). Enhanced coupling can occur within a specific network or across networks. For example, connectivity within the frontoparietal network was reorganized as control-demands increased, reflecting the engagement of rule representation, conflict monitoring, and resolution (Cocuzza et al., 2020; Ray et al., 2020; Nee, 2021). For inter-network connectivity, enhanced functional integration between frontoparietal and attention networks ensures that the information captured by attention can be processed according to the target rules (Cocchi et al., 2013; Cai et al., 2021; Menon and D’Esposito, 2022). In addition, functional integration between frontoparietal and sensorimotor networks ensures that subjects can inhibit dominant responses and behave correctly according to target rules (Cocchi et al., 2013; Cole et al., 2013; Cocuzza et al., 2020). In summary, functional integration is the core neural representations during tasks requiring cognitive control.

Functional integration among the frontoparietal network may be an important neural indicator of the cognitive control ability during aging. In older adults, functional connectivity between prefrontal and parietal cortex, i.e., within the frontoparietal network, positively predicts their performance during tasks requiring cognitive control (Madden et al., 2010). Further, in an emotional working memory task, older adults recruit the frontoparietal network to ignore irrelevant distractors and encode negative emotional items, whereas young adults only adopt the frontal regions to encode both positive and negative emotional items (Ziaei et al., 2017). Similar to young adults, functional integration among the frontoparietal network supports the control functions in healthy older adults. Specifically, frontal regions are thought to engage in rule representation and control execution, whereas parietal lobes are thought to support rule reconstruction (Brass et al., 2005a,b; Ghanavati et al., 2019; Nee, 2021). Thus, decreased functional integration within the frontoparietal network may indicate decreased rule-dependent control in CCA.

Functional integration between frontoparietal network regions (e.g., the dlPFC) and task-specific regions is associated with CCA (as illustrated in Figure 1B). By adding face-based distractors to a delayed-recognition task, Clapp et al. (2011) explored task-based connectivity during working memory performance and observed decreased connectivity between the right dlPFC (called the middle frontal gyrus in their report) and parahippocampus in older adults. Also, during the switch-based task, the functional connectivity between the dlPFC and task-specific regions (e.g., cerebellum, thalamus) was lower in older adults than young people (Madden et al., 2010). In contrast, connectivity preservation between the dlPFC and task-specific regions (e.g., inferotemporal, left premotor cortex) was associated with better cognitive control performance in older adults (Nagel et al., 2011; Hakun et al., 2015). The above evidence suggests that decreased functional integration between frontoparietal network and task-specific brain regions is associated with the decline in cognitive control with age, but the pattern is influenced by tasks or specific subcomponents of cognitive control.

Finally, functional integration, as measured by network efficiency properties, declines with aging. Network integrative processes can be viewed as the communication efficiency measured by graph-theoretic properties (Sporns, 2013). In addition to the strength of functional connectivity, graph-theoretic coefficients such as rich clubs and hub integrity can be used to reflect the functional integration. The assortativity, a graph coefficient that reflects how regions connect to other regions with a similar degree, was lower in older adults during control tasks than at rest, indicating an aging-related decline in functional integration (Grady et al., 2016). In contrast, preserved functional integration was associated with better cognitive control performance. Specifically, both higher hub integrity of the dlPFC and anterior cingulate cortex (Pa et al., 2014), and greater integration of the dorsal attention network predicted better inhibitory control ability in older adults (Rieck et al., 2021). The graph-theoretic analysis offers an additional approach to elucidate CCA-related changes in functional integration.

### Disrupted inter-network functional antagonism

Cognitive control relies on functional antagonism between large-scale networks. Functional antagonism differs from functional segregation. Functional segregation usually refers to positive functional connectivity, where blood-oxygen dynamics between regions are predominantly positively correlated, whereas functional antagonism refers mainly to negative connectivity, where the blood oxygen level-dependent signals between paired regions are predominantly negatively correlated (Fornito et al., 2012; Li et al., 2021; Demertzi et al., 2022). Functional antagonism can exist in both resting and task states and is mainly present between the TPN and DMN. In the resting state, the TPN is usually only slightly activated, while the DMN is highly activated, and there is a stable negative connection between the TPN (e.g., attention and frontoparietal network) and DMN (Raichle, 2015; Buckner and DiNicola, 2019). In a cognitive control task, the activation level of the DMN is decreased, while that of the TPN is increased; thus, the functional antagonism between these networks is maintained. Functional antagonism between the TPN and DMN positively predicts cognitive control performance in healthy adults (Kelly et al., 2008; Xin and Lei, 2015).

Declines in functional antagonism are important neurological indicators of the decreased cognitive control in older adults (as illustrated in Figure 1C). There is ample evidence that functional antagonism between the TPN and DMN decreases with aging, as evidenced by the decreased anti-correlation between activity in the DMN and in the ventral and dorsal attention network (Geerligs et al., 2015; Ferreira et al., 2016; Spreng et al., 2016; Zonneveld et al., 2019). Furthermore, even in healthy older adults, a large number of connections shift from negative to positive, implying impaired functional antagonism in the aging brain (Ferreira et al., 2016). Putcha et al. (2016) found that decreased functional antagonism between attention networks and the DMN was associated with cognitive control decline in older adults. The TPN antagonism supports the appropriate suppression of the DMN during a range of cognitive control tasks, which ensures that the external-oriented cognitive process is not affected by internal processes from the DMN (Raichle, 2015; Buckner and DiNicola, 2019). Thus, decreased DMN–TPN antagonism putatively indicates that the decrease in CCA is associated with enhanced internal inference from the DMN.

### Summary

Aging-related changes in the functional connectivity of large-scale networks are important contributors to CCA. Specifically, decreased functional segregation, integration, and antagonism are associated with worse behavioral performance measured by control-based tasks in older adults (Madden et al., 2010; Putcha et al., 2016; Setton et al., 2021). Despite the debilitating effects of disrupted network connectivity, there is also evidence that functional integration is positively correlated with task performance in older adults (Nagel et al., 2011; Hakun et al., 2015). Thus, connectivity-related compensation, especially in terms of functional integration, could be examined in future studies. Functional segregation in the resting state is a hallmark of the well-organized spontaneous network organization, functional integration in the task state reflects collaborative processing of information, and functional antagonism ensures resistance to endogenous interference during cognitive control (Cocchi et al., 2013; Sporns, 2013; Wig, 2017; Buckner and DiNicola, 2019). Aging is accompanied by abnormalities in all three types of network interaction patterns, suggesting that decreased CCA is associated with disrupted spontaneous network organization, impaired information coordination, and increased endogenous interference. In both resting and task states, changes in the intra- and inter-connectivity of the frontoparietal network states play a central role in decreasing CCA (Madden et al., 2010; Sala-Llonch et al., 2012; Setton et al., 2021). Finally, it is known that maintenance of functional connectivity depends on structural connections, consisting of white matter fibers. How does the white matter structural network change with age? How do functional and structural networks interact with and participate in CCA? In the next section, we introduce structural evidence to elucidate the network connectivity mechanisms related to CCA further.

## Structural and functional network interactions in cognitive control in aging

Anatomically, a large number of isotropic axons are “bundled” together to form white matter fibers. These fibers form a structural network that ensures the transmission of electrical signals across brain regions (Bennett and Madden, 2014; Liu et al., 2017). In healthy adults, although some functional connections could exist without direct structural support (Zimmermann et al., 2016), several studies have reported that the strength, length, and spatial position of white matter fibers can predict functional connectivity in resting and task states (Honey et al., 2009; Hermundstad et al., 2013). White matter networks may act as “skeletons” that maintain and constrain functional connectivity (Park and Friston, 2013; Suárez et al., 2020). Aging is usually accompanied by white matter structural lesions, manifested by decreases in white matter volume and fiber disconnection (Bennett and Madden, 2014; Damoiseaux, 2017). These structural declines trigger alterations in functional connectivity, causing older adults to exhibit declines in cognitive control at the behavioral level. In this section, we discuss the neural basis of the multimodal network mechanisms underlying decreased cognitive control in older adults, based on the limited empirical studies available to date.

### Age-related changes in white matter and cognitive control in aging

Age-related structural changes in the white matter involve both decreases in white matter volume and impairment of structural connections (Damoiseaux, 2017; Farokhian et al., 2017). Voxel-based morphometry analysis of T1-weighted images revealed a 26% decrease in the total white matter volume from the age of 30 years to the age of 90 years (Jernigan et al., 2001). Compared to the occipital and temporal lobe regions, the frontal white matter in the anterior hemisphere is the most vulnerable to age-related loss due to aging (Gunning-Dixon et al., 2009). In addition, several studies have analyzed diffusion tensor imaging and diffusion-weighted imaging using white matter fiber-tracking techniques to examine the relationship between structural connectivity and aging. These results showed that the structural connectivity of the cingulate gyrus, cuneus, precuneus, superior frontal gyrus, and parietal lobe gradually decreased with age, suggesting brain-wide structural connectivity impairment (Madden et al., 2012; Hirsiger et al., 2016; Damoiseaux, 2017; Liu et al., 2017).

Both white matter loss and structural disconnection suggest a decrease in neural signaling capacity and are associated with decreased cognitive control (Charlton et al., 2010; Ystad et al., 2011; Madden et al., 2012). In older adults, volume loss in the frontal, inferior frontal, and parietal white matter predicts declines in multiple subcomponents of cognitive control, including working memory, inhibitory control, and cognitive flexibility (Charlton et al., 2010; Madden et al., 2012). In addition, lesions of the superior longitudinal fasciculus can predict cognitive flexibility decline in older adults (Gold et al., 2010). Notably, white matter lesions associated with decreased CCA are mainly concentrated in the frontal and parietal lobes (Charlton et al., 2010; Madden et al., 2012), which overlap spatially with the frontoparietal network. In addition, structural connectivity between subcortical regions (i.e., the thalamus and nucleus accumbens) and regions within the DMN and dorsal attention network positively predicts cognitive control in older adults (Ystad et al., 2011). The structural connectivity between the thalamus and pre-supplementary motor region is also positively correlated with inhibitory control in older adults (Coxon et al., 2012). In summary, both aging-related regional and connectivity damage to the white matter play a role in the decline of cognitive control with age.

### Multimodal networks involved in cognitive control in aging

**FIGURE 2:**
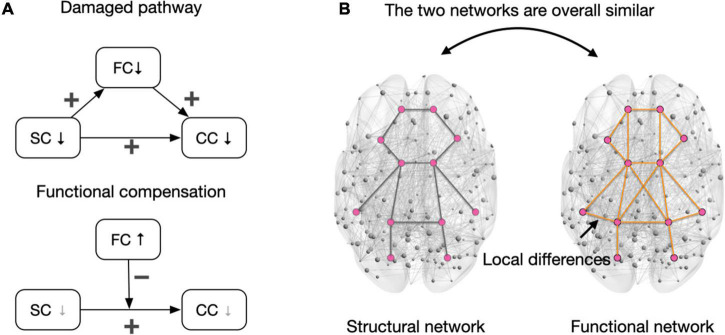
Interactions of the functional and structural network in cognitive control. **(A)** The upper panel depicts the damaged pathway of “structural connectivity damage → functional connectivity damage → cognitive control decline” when the structural network damage is severe. Notably, the direct path (SC→CC) indicates a general relationship between decreased structural connectivity and cognitive control decline, and there may be other mediating variables beyond functional connectivity in maintaining this relationship. In the bottom panel, the gray downward arrow in SC indicates that the structural network is relatively intact or slightly damaged. In this case, the compensation of functional connectivity reduced the debilitating effects of structural connectivity damages on cognitive control. The gray downward arrow in CC indicates no decline (especially when the task is easy) or fewer declines in cognitive control when functional connectivity is compensated. The presentation of variable relationships followed the conventions in the multivariate model. **(B)** The structural and functional networks are generally similar, but functional connections can be built on tripartite structural pathways to retain connectivity (Suárez et al., 2020). This pattern (i.e., the structural and functional network are similar in whole but different in part) can be applied to the whole brain network. A relatively intact structural network is a prerequisite for compensating for functional connections. Pink dots represent brain areas, and lines between regions represent connections. SC, structural connectivity; FC, functional connectivity; CC, cognitive control; ↓, decrease; ↑, increase; +, positive relationship between two linked variables; –, increased FC diminishes the effect of structural connectivity damage in triggering cognitive control declines.

Functional networks may serve as mediators of the cognitive control decline triggered by structural network damage. The impairment perspective proposes that damage to structural connections can weaken functional connectivity. Studies have shown that structural connections support functional connectivity in healthy older adults. For example, Andrews-Hanna et al. (2007) found that fractional anisotropy (a measurement of white matter integrity) of the anterior–posterior fiber was positively correlated with the functional connectivity between hub regions within the DMN, including the medial prefrontal cortex and PCC. Other studies have also shown that the integrity of the fornix and cingulum bundle can positively predict functional connectivity (e.g., cortex–subcortical connectivity) (Kehoe et al., 2015; Fjell et al., 2016). Not surprisingly, damage to structural connections results in reduced functional connectivity. A longitudinal study suggested that age-related impairments in the structural connectivity of the cingulum bundle trigger decreased cortical–subcortical functional connectivity (Fjell et al., 2016). Since functional connectivity supports the collaborative processing of information in control tasks, the pathway “structural connectivity damage → functional connectivity damage → cognitive control decline” may be a valid concept in terms of CCA (Figure 2A, damaged pathway).

However, the damage perspective cannot explain some of the existing empirical results and may lead to a view of functional connectivity compensation (Figure 2A, functional compensation). Specifically, while lesions in structural connectivity can trigger cognitive control decline in older adults, increased functional connectivity can attenuate cognitive decline associated with structural damage. Benson et al. (2018) reported that increased functional connectivity of the frontoparietal and attention networks counteracted cognitive control declines associated with white matter lesions. Therefore, white matter lesions do not necessarily trigger functional connectivity impairment. In older adults, the TPN can maintain cognitive function as much as possible by enhancing functional connectivity. Why do structural connectivity lesions in the aging brain not necessarily weaken functional connectivity? What is the structural basis of compensatory enhancement of functional connectivity? The possible explanation of above questions is discussed in the following paragraph.

A relatively intact structural network may be a prerequisite for functional connectivity compensation, while severe lesions in the structural network can trigger reduced functional connectivity. When considering a network as a whole, a marked similarity between functional and structural networks can be observed (Figure 2B; Wang et al., 2015; Suo et al., 2021). Structure–function coupling reflects the support and restraint of structural networks on functional networks (Suárez et al., 2020; Bazinet et al., 2021; Suo et al., 2021). However, structural and functional connections do not strictly correspond to each other, which reflects the adaptive adjustments of specific connections in the human brain (Suárez et al., 2020). On the other word, functional connectivity can be built on third-party structural pathways. For older adults, when the structural network is relatively well preserved, lesions of specific structural connections do not directly result in damage to functional connectivity, and the preserved third-party structural pathways ensures the keep of functional connectivity. However, severe structural network damage makes it difficult to rely on third-party structural pathways to maintain the initial functional connectivity and results in cognitive control decline in older adults. For example, Burianova et al. (2015) selected hyperactivated regions in working memory tasks to define the compensatory network and found that integrity of the bilateral frontoparietal tracts positively predicted the strength of functional connectivity of the compensatory network as well as task performance. Notably, although functional connectivity can be maintained when the structural network is relatively intact, the functional connectivity built on a third-party structural pathway may have reduced communication efficiency due to the increased length of the structural route. This can be explained using a metaphor: when the direct road to the destination is blocked, one can take a detour to get to the destination, but the total distance is longer. The aging brain may recruit more neural synchronization to offset the reduced communication efficiency and manifest as increased functional connectivity.

### Summary

Structural and functional networks show complex interactions affecting CCA. Because structural connections support and constrain functional connections, it could be concluded that structural connection damage triggers cognitive control decline by weakening functional connectivity. However, when the structural network is locally damaged but globally preserved, the effects on functional connectivity may be compensated through third-party structural pathways and may counteract the cognitive control decline associated with the structural damage. Functional compensation determined by preservation of the structural network should be considered in future explorations of network mechanisms underlying CCA.

## Prospects and limitations

Aging-related changes in structural and functional connectivity among networks are involved in decreased CCA. However, to date, CCA-related network mechanisms have been established based on correlative evidence (Ferreira and Busatto, 2013). In the strict sense, we cannot specify whether changes in network connectivity caused cognitive control declines in older adults. Causal evidence needs to be accumulated to increase the reliability of existing findings. Notably, several prospects are available for the network mechanism of CCA, as discussed below.

### Exploring the shared and unique network mechanisms underlying cognitive control in aging

Common and unique network mechanisms underlie cognitive control. Cognitive control can be divided into three core subcomponents: working memory, inhibitory control, and cognitive flexibility (Diamond, 2013; Zink et al., 2021). All three core subcomponents are based on top–down control but have unique cognitive mechanisms. For example, working memory is related to the representation and extraction of information, whereas inhibitory control is mainly associated with reducing interference and highlighting the target (Diamond, 2013). Imaging evidence suggests that the three subcomponents rely on the frontoparietal network to implement conflict monitoring, resolution, and goal-directed processes (Cocchi et al., 2013; Zanto and Gazzaley, 2013; Qiao et al., 2020). However, the frontoparietal network dynamically rewires functional connections with regions involved in other networks according to task rules, forming component-specific network mechanisms (Cole et al., 2013; Zanto and Gazzaley, 2013). For example, working memory involves increased functional connectivity between the frontoparietal network and memory-related brain regions (Yamashita et al., 2018; Cai et al., 2021), while responsive control is associated with enhanced functional connectivity between the frontoparietal and sensorimotor networks (Cocchi et al., 2013).

Aging-related network mechanisms underlying the different subcomponents of cognitive control may be both shared and unique. Declines in functional connectivity within the frontoparietal network are associated with decreased CCA (Sala-Llonch et al., 2012; Setton et al., 2021); however, compensatory enhancement of functional connectivity within the network counteracts the adverse effects of aging (Benson et al., 2018). In addition, white matter lesions within the frontoparietal network predict declines in multiple aspects of cognitive control (Charlton et al., 2010; Madden et al., 2012). Multimodal imaging evidence points to common mechanisms underlying CCA in the frontoparietal network. Moreover, aging-related changes in inter-network connectivity show task-specific patterns, suggesting the existence of unique network mechanisms related to CCA. Notably, previous studies used different MRI data pre-processing protocols, and network construction and functional connectivity analysis methods (Stanley et al., 2015; Tsvetanov et al., 2018); therefore, it is impossible to compare results across studies directly. In future, datasets from the same cohort of older adults, addressing multiple cognitive control components, will be needed to explore this issue.

### Enhancing multimodal network studies in cognitive control in aging

Multimodal network mechanisms for CCA are mostly theoretical, and empirical studies are rare. The impairment perspective suggests that CCA follows the pathway of “structural connectivity impairment → functional connectivity impairment → cognitive control decline.” However, the compensation view suggests that if the structural network is mostly preserved, the compensation of functional connectivity can be occurred through third-party structural pathways, as manifested by increased functional connectivity and cognitive control maintenance. When the structural network is severely disrupted, the functional network will be damaged and result in declines in cognitive control. Thus, structural and functional networks act together in a complex way in CCA, and the degree of structural network impairment may be an important a *priori* variable. In future, researchers should systematically collect multimodal imaging data, select targeted connections or networks, and construct mediating and moderating multivariate models to clarify how structural and functional networks interact and influence CCA.

Notably, communication models and multilayer network analyses that emerge from network science would offer approaches to explore the structure–function coupling of brain networks (Bassett and Sporns, 2017). By formulating models of multimodal connectivity, these methods hinder the non-independence of multilevel connectivity measures and assess the extent to which the biologically realistic model conforms to the properties of the functional network (Kivela et al., 2014; Muldoon and Bassett, 2016; Bassett and Sporns, 2017; Suárez et al., 2020). These methods can characterize the similarity of structural and functional networks at the macro level and will allow an integrated assessment of the supporting and constraining effects of structural networks on functional networks, thereby offering a new approach for exploring multimodal network mechanisms underlying CCA.

### Exploring the dynamic functional connectivity mechanisms of cognitive control in aging

Whether changes in dynamic functional connectivity are involved in decreased CCA should be examined. Dynamic functional connectivity refers to the time-varying fluctuations of functional networks (Hutchison et al., 2013; Allen et al., 2014; Calhoun et al., 2014). Cognitive control has been found to be associated with dynamic functional connectivity (Hutchison and Morton, 2016; Nomi et al., 2017). For example, Nomi et al. (2017) found that the frequency of an asynchronous brain state was associated with control-based tasks that require flexible cognition. Besides, aging-related changes in dynamic functional connectivity have been verified (Cabral et al., 2017; Viviano et al., 2017). Nevertheless, investigations of CCA have largely taken into account the connectivity properties from a static perspective. Based on the available studies, it is difficult to form specific conclusions about whether and how aging-related changes in dynamic functional connectivity are involved in decreased CCA. Hence, dynamic approaches of functional connectivity, such as sliding-window methods (Hutchison et al., 2013), should be adopted in future studies.

## Conclusion

Cognitive control decline is a salient feature of aging. Abnormalities in the functional segregation, integration, and antagonism of functional networks suggest that disrupted spontaneous network organization, failed information co-processing, and increased endogenous interference in cognitive control are related to reduced CCA. Severe damage to the structural network can induce cognitive control decline by weakening functional connectivity. Nevertheless, a relatively intact structural network can ensure the compensation of functional connectivity, to delay the decline in CCA. Future research should introduce network neuroscience approaches and investigate the multimodal network mechanisms of aging-related cognitive control decline.

## Author contributions

HX: writing and revising the article, drafting, and proofreading references. QH and AC: reviewing and revising the manuscript and giving critical comments. All authors contributed to the article and approved the submitted version.
